# Decoding event-related potentials: single-dose energy dietary supplement acts on earlier brain processes than we thought

**DOI:** 10.3389/fninf.2025.1563893

**Published:** 2025-07-08

**Authors:** Karina J. Maciejewska

**Affiliations:** Institute of Biomedical Engineering, Faculty of Science and Technology, University of Silesia in Katowice, Katowice, Poland

**Keywords:** decoding brain’s function, EEG, event-related potentials, multivariate pattern analysis, attention

## Abstract

**Introduction:**

This paper describes an experimental work using machine learning (ML) as a “decoding for interpretation” to understand the brain’s physiology better.

**Methods:**

Multivariate pattern analysis (MVPA) was used to decode the patterns of event-related potentials (ERPs, brain responses to stimuli) in a visual oddball task. The ERPs were measured before (run 1) and after (30 min—run 2, 90 min—run 3) a single dose of an energy dietary supplement with only a small amount of caffeine.

**Results:**

Its effect on ERPs was successfully decoded. Above-chance decoding accuracies were obtained between ∼350 and 450 ms (corresponds to P3 peak) after stimulus onset for both the placebo and study groups, whereas between ∼200 and 260 ms (corresponds to P2 waveform) only in the placebo group. Moreover, the decoding accuracies were significantly higher in the placebo than in the study group in the 200–250 ms and 450–500 ms time bins. Our previously reported findings showed an increase in P3 amplitude among the runs only in the placebo group, indicating a reduction of mental fatigue caused by the supplementation.

**Discussion:**

Thus, this paper extends these results, showing that the dietary supplement affected the brain’s neural activity related to the attention-related processing of the visual stimuli in the oddball task already at the early processing stage. This implies that inhibiting the fatigue-related brain changes after only a single dose of a dietary neurostimulant acts on early and late processing stages. This emphasizes the value of decoding for interpretation in ERP research. The results also point out the necessity of controlling the uptake of dietary supplements before the neurophysiological examinations.

## 1 Introduction

The high speed of life in these times increases the cognitive demands on our mind and body. Therefore, seeking external psychostimulants that boost our energy level, vigilance, and performance is natural. Probably the most studied one is caffeine, though other substances, such as taurine, vitamin B, guarana, yerba mate, carnitine, and ginkgo biloba, have similar effects (Bäcker and Jaitner, 2023; Buckenmeyer et al., 2015; Daou et al., 2019; Glade, 2010; Pradhan and Pal, 2020; Wassef et al., 2017). Many products on the market are rich in these substances and easily accessible for everyone, e.g., energy drinks. Therefore, the majority of the studies focus on the adverse effects of such products, which, due to high doses, possible toxic interactions, and/or long-term use, may result in severe health issues. They became a significant concern for health providers around the world (Cao et al., 2021; Costantino et al., 2023; Higgins et al., 2010, 2015; Kaur et al., 2022; Mendoza et al., 2023; Surma et al., 2023).

On the contrary, typical moderate caffeine intake is considered safe, and smaller doses of neurostimulants have been shown to improve cognitive performance (Hajsadeghi et al., 2016; Turnbull et al., 2017). The reported beneficial effects of caffeine-based and multi-ingredient supplements relate to increased wakefulness, alertness, energetic arousal, mood, improved psychomotor and mental performance (e.g., memory, attention, choice reaction, and concentration), decreased mental fatigue and stress, and reduced levels of amino acids linked to cognitive impairment, mental disorders (Bäcker and Jaitner, 2023; Buckenmeyer et al., 2015; Daou et al., 2019; Glade, 2010).

One of the most powerful techniques to study cognitive processes is electroencephalography (EEG) due to its excellent temporal resolution, allowing researchers and clinicians to reveal neural processes in healthy and pathological conditions (Cohen, 2014; Luck, 2014). In addition, when the participants are presented with visual, auditory, or somatosensory stimuli during EEG acquisition, the brain’s neural responses elicited by these stimuli are analyzed through the specific EEG signal patterns, time-locked to the stimulus (event-related potentials, ERPs). The pattern of the recorded brain activity is an estimate of the particular neurocognitive processes, such as attention, working and long-term memory, or spatial orientation, measured under experimental manipulation, which can be used to study the impact of neurostimulants (Cohen, 2014; Luck, 2014).

Attention is one of the most important cognitive mechanisms. It acts at an early, perceptual, as well as at the later, post-perceptual level. Thus, ERPs are a perfect method to study attention, as they can dissociate the processing stages and types of attentional processes. The early effects are related to visuospatial attention, observed as a P1 wave suppression for the stimuli at unattended locations (first positive ERP, beginning 70–100 ms after stimulus onset), followed by an N1 waveform (first negative ERP) enhancement at attended locations. The P1 attention effect reflects a suppression of feedforward sensory processing at unattended locations. On the other hand, the N1 attention effect reflects the operation of a limited-capacity discrimination process directed to the information presented at the attended location. However, attentional mechanisms can be modulated by features other than location. This type of selection based on non-spatial features (featural attention) is mostly observed as a broad posterior effect (selection negativity) and the more temporally discrete anterior effect (anterior P2 attention effect, anterior selection positivity). Different non-spatial dimensions have been shown to elicit feature-based attention, like color, orientation, form, or direction of motion. When perceptual systems are overloaded, attention operates at an early stage and influences the early sensory ERP components. However, selective attention may also operate on post-perceptual processes when the stimuli overload memory encoding or response selection systems. The P3 component is a typical late ERP elicited in an oddball paradigm, where there are at least two stimulus categories, of which one contains rare (targets) and the other contains frequent stimuli (standards). P3 amplitude is higher for the infrequent task-relevant events and can be influenced by the amount of attention allocated to a stimulus, whereas its latency represents the classification speed (Luck, 2014; Luck and Kappenman, 2012).

However, studies investigating the impact of low-dose energy boost supplements on objective measures of brain activity are scarce. There are studies showing that even low doses of caffeine and/or other neurostimulatory substances may improve cognitive performance, but they are mostly based on behavioral data (Haskell-Ramsay et al., 2018; Kennedy et al., 2008; Smit and Rogers, 2000). Only a few studies investigated the impact of lower doses of caffeine on EEG or ERPs (Ajjimaporn et al., 2022; Meng et al., 2017; White et al., 2017). Ajjimaporn et al. studied the acute effect of a 50 mg caffeinated drink (CAF) on resting state EEG (rsEEG) and other measures of cognitive and physical performance. The authors observed diminished alpha wave activity and improvement of cognitive function on working memory following caffeine consumption. Meng et al. investigated the effects of soft drinks and regular coffee on rsEEG and on the performance of motor imagery-based brain-computer interface (BCI). The authors observed a decrease in alpha and beta power after caffeine consumption compared with control and sugar conditions. However, there are several limitations of these studies, e.g., lack of proper control conditions. In addition, White et al. (2017) studied the effect of a single dose of multivitamin and mineral combinations with and without guarana on functional brain activity during a continuous performance task. The Authors concluded that their results suggested that a single multivitamin/mineral dose was sufficient to impact functional brain activity in task-related brain regions.

Therefore, this work is a part of our project on the influence of a single dose of a dietary supplement with a small amount of caffeine (55 mg) on EEG and ERPs in healthy participants. Earlier, we found an increase in P3 amplitude throughout the experimental session only in the placebo group, whereas it remained at the same level in the study group. This effect was related to the increased number of attentional resources allocated to perform the task due to increasing mental fatigue, which was inhibited in the study group (Maciejewska and Grabowska, 2020). More sophisticated approaches have extended the classical univariate analysis due to their higher sensitivity, e.g., non-parametric cluster-based permutation analysis (Maris and Oostenveld, 2007).

However, multivariate decoding methods may be even more advantageous for studying brain functions (Hebart and Baker, 2018). Using machine learning (ML) in data analysis of time series has a rich history. However, it has been mainly used to extract features of the signal in biofeedback closed-loop applications like BCIs, e.g., to steer a wheelchair or prosthesis. Other typical applications are classification methods used in data mining in diagnostics and treatment for automatic classifications of physiological and pathophysiological signals (Gao et al., 2018; Lopez-Garcia et al., 2018; Salas-Gonzalez et al., 2010; Wall et al., 2012).

Interestingly, in addition to this standard approach—“decoding for predictions” in real-world applications (Hebart and Baker, 2018), there is another line of ML applications in signal analysis, which is of special significance for neuroimaging. In recent years, there has been growing attention to using multivariate “decoding for interpretation” to understand the human brain better (Grootswagers et al., 2017; Hebart and Baker, 2018). Due to its higher sensitivity, using multivariate pattern analysis (MVPA) may increase the statistical power of the study, which in turn results in finding possible weaker effects that might not be captured by the conventional methods. Conventional ERP analyses ask whether the difference in voltage between the studied conditions (averaged across participants) is large relative to the variability across participants. Anything that causes variability across participants, including differences in brain folding that cause differences in scalp distribution, reduces the likelihood that the true difference in brain activity between the two conditions is statistically significant. On the other hand, decoding is done separately for each subject, and then a t-test is used to ask whether decoding accuracy at each time point is significantly better than chance. It does not matter whether different subjects have different scalp potential distributions. This tends to minimize the standard error, giving us larger *t*-values than in the conventional analysis. Therefore, decoding is usually more sensitive than the traditional approach (i.e., decoding gives greater statistical power) (Carrasco et al., 2024; Luck, 2023). ML has been used to decode ERPs in several cognitive processes, e.g., working memory, attention, motion direction, face perception, expectation violations, and even personality traits (Bae, 2021; Bae and Luck, 2018, 2019a; Jach et al., 2020; Lowe et al., 2023; Maciejewska and Froelich, 2021; Turoman et al., 2024; Valeriani et al., 2001).

This work has three aims. The first one was to investigate further the acute effect of energy boost supplementation on processing visual stimuli in an oddball task. To this end, MVPA was performed to better discriminate the ERPs recorded before and after the digestion of a single dose of a caffeinated dietary supplement. Second, the ML results were compared with the results of univariate tests, as well as the non-parametric cluster-based permutation analysis obtained by us earlier (Maciejewska and Grabowska, 2020). Finally, to evaluate the influence of the classification parameters setup, the classifier performance was compared among three sets of decoding parameters: (1) the minimal required - 10 trials per ERP average and 3 cross-validation blocks, which resulted in including 31 participants, (2) 13 trials per ERP average and 3 cross-validation blocks (24 participants included), and (3) 10 trials per ERP average and 4 cross-validation blocks (24 participants included).

Decoding offers greater sensitivity compared to conventional methods. In the standard approach, the ERP measures are analyzed using the classical univariate analysis, i.e., by statistical testing significant differences in ERP amplitudes or latencies at the group level (by comparing mean ERP amplitude calculated for a pre-defined time window, averaged across all stimulus repetitions) or time point by time point, as is done in the cluster-based permutation analysis). Instead of comparing grand-averaged ERPs (i.e., mean ERPs calculated across all the participants), decoding works at the single-subject level. Since decoding is done separately for each participant, no assumption of the same scalp distributions is necessary. Therefore, in this work, MVPA is expected to reveal subtle changes in neural processing underlying the studied brain functions that more conventional methods have failed to detect.

The significance of this work lies in investigating the possible beneficial effect of only a single dose of a dietary supplement with much less caffeine than a cup of coffee on brain physiology in mental fatigue. However, the studied problem is also important in the neurophysiological examinations, where controlling the uptake of such supplements might be crucial. In addition, the results allow translation into clinical applications focused on pathophysiology related to attention, anxiety, mood, and memory disorders.

## 2 Materials and methods

### 2.1 Participants

This work does not contain any data collection, as it presents the results of an analysis performed on previously reported data (Maciejewska and Grabowska, 2020). In the original experiment, healthy volunteers were examined in a double-blind, placebo-controlled study. Forty-seven young, healthy, physically active students or university alumni (27 women, according to the questionnaire) at the age of 26.1 ± 4.6 years were recruited for the experiment. The sample size has been determined based on the previous work and is typical in ERP research (Bae, 2021; Bae et al., 2020; Bae and Luck, 2019b; Grootswagers et al., 2017; Michalkova et al., 2022). All the participants were right-handed, moderate caffeine users, had normal or corrected to normal visual acuity, and had no history of neurological or psychological disorders. None of the participants had consumed alcohol, coffee, intoxicants, energizing beverages, or other such substances within at least 12 h before the study. The study was conducted with the understanding and written consent of each subject, following The Code of Ethics of the World Medical Association, the Helsinki Declaration of 1975, as revised in 2000, and has been approved by the Committee of Ethics of the University of Silesia in Katowice on scientific studies conducted on humans (number 2/2018) which could be provided upon request.

In a typical ERP analysis, it is recommended to perform the power calculation to ensure the sample size is sufficient to reliably evaluate the studied effects. The expected effect size for this study was medium (η*_*p*_*^2^ = 0.06). The *post hoc* power calculation, performed using G*Power v. 3.1 (Faul et al., 2007) for the alpha level of 0.05, gave a power of 0.85 (which is considered strong power, above the standard threshold of 0.80) for a total sample size of 30, and a power of 0.75 for 24 participants, suggesting a good chance (75%) of detecting a true effect. These calculations indicate that our sample size (i.e., the number of participants) included in the analyses is sufficient to detect the expected effects. However, one must bear in mind that such calculations are performed for standard statistical tests, where the measures of central tendency (such as the mean or median of all data points in each group) are compared among the conditions. On the other hand, decoding benefits from operating at the single-subject level, which makes it more sensitive than the conventional approach (Carrasco et al., 2024). Therefore, an even smaller sample size might be sufficient to find a true effect using decoding.

### 2.2 Experimental procedure

The original study aimed at studying the effect of a single dose of caffeinated energy boost dietary supplement dissolved in a cup of water (with a total amount of 55 mg of caffeine, including caffeine extract, guarana, yerba mate, cocoa powder, vitamin B and other vitamins and minerals) on ERPs in a visual oddball paradigm. A double-blind, placebo-controlled study was used, where neither the participants nor the data collectors knew which group the participant belonged to. Half of the participants (the study group) received the active substance. The other half (the placebo group) received a placebo designed to appear, as much as possible, like the active substance. They drank one cup of water with dissolved one effervescent tablet containing vitamin C in a similar dose as was in the dietary supplement. The drinks in both groups had the same taste, smell, and color.

EEG was recorded in three runs: before the supplementation (run 1), 30 min after the supplementation (run 2), and 90 min after the supplementation (run 3). The participants were instructed to gaze at the center of the black screen during interstimulus intervals and observe the stimuli (yellow font on a black background): letters (frequent stimuli, standards) and digits (rare stimuli, targets) presented in the center of the computer monitor. The stimulus parameters were: 200 ms duration and 1,000 ± 200 ms interstimulus interval. The stimuli were presented in random order for each participant in each run. Their probabilities were: 20% for the target and 80% for the standard stimuli. The overall number of stimuli was 250 (200 standard and 50 target stimuli), and participants were instructed to push a pad button after each target stimulus. Details of the study design were previously described in Maciejewska and Grabowska (2020).

### 2.3 EEG acquisition

The EEG signal was recorded from 32 Ag/AgCl electrodes embedded in an elastic Waveguard™ EEG cap in the extended 10/20 EEG montage system with AFz electrode as the ground electrode, common average reference, and 256 Hz sampling rate. The EEG signal was converted to a digital time series and amplified using an ANT Neuro amplifier (AMP-TRF40AB model) in DC with a 20,000-amplification gain. The Advanced Source Analysis system, ASA-Lab (ANT Neuro), with ASA v.4.8 software, was used for acquisition. No filter was applied during data acquisition, except for the anti-aliasing low-pass filter with cutoff frequency = 0.2* sample frequency (51.2 Hz for the sampling frequency of 256 Hz).

### 2.4 EEG pre-processing

**FIGURE 1:**
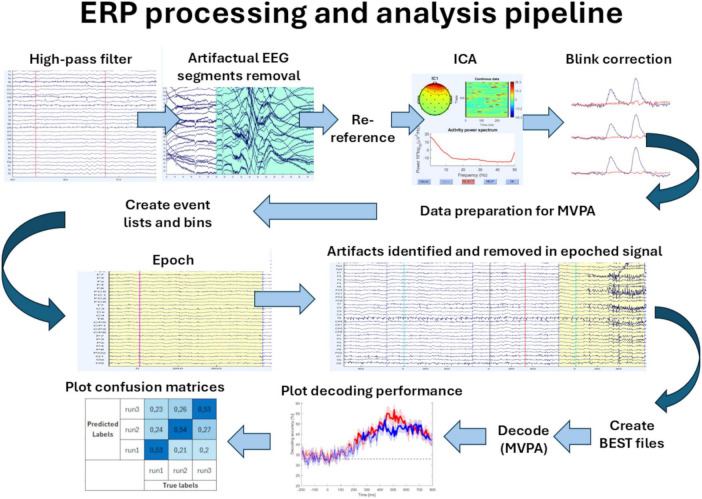
ERP pre-processing and analysis pipeline. The ERP pre-processing included: high-pass filtering, visual inspection and removal of the artifactual segments, re-referencing, ICA decomposition, blink artifact correction, data reorganization for MVPA, creating event lists and bins, epoching, and artifact removal in the epoched data. The MVPA analysis included: creating Extract Bin-Based Single Trials (BEST) files (to convert the epoched datasets into a format that is more convenient for pattern classification), decoding using MVPA (multivariate pattern classification), plotting the decoding performance, and plotting the confusion matrices.

EEG pre-processing and analysis were performed in MATLAB R2021b (Mathworks) with open-source MATLAB packages for M/EEG analysis: EEGLAB (Delorme and Makeig, 2004) and ERPLAB 10.04 (Lopez-Calderon and Luck, 2014). During offline pre-processing, the recorded EEG signal was filtered using a high-pass non-causal Butterworth filter [with 0.1 Hz half-amplitude cutoff and 12 dB/octave slope (second-order)]. Large artifacts, seen as high-amplitude voltage deflections in many channels (not specific to any particular channels but usually occurring in most of them), other than typical eye or muscle artifacts, and mostly related to the movement of the participants, were rejected manually during visual inspection. The signal was re-referenced to the average of the mastoids. Independent component analysis (ICA) using EEGLAB’s runica algorithm was used for eye blink artifact correction based on: the anterior scalp distribution, the location in the ERP image, and the spectral histogram of independent components (ICs). This resulted in identifying one or two components for each participant. Continuous signal was then epoched into 1-s long time windows, starting 200 ms before the stimulus (baseline). The choice of the epoch length was made because the P3 waveform (the latest ERP elicited in this paradigm) peaks around 400 ms, and this length is commonly used to study this component, as well as to maintain integrity with the results obtained before. The time points used for the analysis were consistent across all trials for each subject. Moreover, the fewer time points, the better for the MVPA. Trials with incorrect behavioral responses or eye blinks occurring during stimulus presentation (i.e., from −200 ms before to 200 ms after the stimuli) were excluded from the averages. The mean number of target stimuli after these steps of data processing was 48 ± 3. Data acquisition and the first pre-processing steps described above were done in our previous, original study (Maciejewska and Grabowska, 2020). The following preprocessing steps that were needed to prepare the data for the upcoming decoding were done in this work: renaming the trial types, merging the datasets, creating new eventlist files, assigning the trials to the bins, re-epoching, rejecting epochs that contained signal boundaries, and artifact rejection using ERPLAB’s moving window peak-to-peak threshold tool. Trials in which peak-to-peak voltage exceeded 100 μV in 200 ms time windows (with 50 ms window step) were detected and excluded from the analysis. Then, the datasets were used in the MVPA described below, which was entirely conducted in this work. The pipeline presenting the pre-processing and analysis steps is shown in Figure 1.

### 2.5 Multivariate pattern analysis of ERPs

The multivariate pattern analysis was performed using ERPLAB’s Multivariate Pattern Classification Tool (Lopez-Calderon and Luck, 2014), designed to perform decoding of the ERPs. To this end, the classifier was trained to distinguish the classes of stimuli based on the pattern of voltage scalp distribution based on the EEG signal measured from scalp channels (Bae, 2021; Bae and Luck, 2018, 2019a; Grootswagers et al., 2017; Luck, 2023). The goal of this study was to determine whether the ERP signal contains information about the experimental run it came from, i.e., before the supplementation (run 1), 30 min after the supplementation (run 2), and 90 min after the supplementation (run 3), using above-chance decoding accuracy as the most straightforward evidence that such information is present (Bae and Luck, 2018). Since the goal of decoding was to find the neurophysiological effect of the experimental manipulation (supplementation) through the changes in the ERP patterns recorded throughout the experimental session, elicited by the task-relevant stimuli, the decoding was performed on the epochs elicited by the target stimuli. Keeping in mind three facts: (1) P3 is a robust, well-known ERP elicited in a variety of oddball experiments, (2) knowing its parameters, and (3) having analyzed the ERPs elicited in this experiment using standard ERP techniques in our previous works, there was no reason to decode the ERPs elicited by the standards from the ERPs elicited by the targets. Moreover, the research question asked in this study was whether the ERPs elicited by the task-relevant targets contain information about the experimental run.

MVPA, also referred to as decoding or classification (Chadwick et al., 2012; Weaverdyck et al., 2020), is a class of methods (a type of analysis) that is used to distinguish the classes of stimuli based on the pattern of voltage scalp distribution from the EEG signal measured from scalp channels. Here, the classes were the three runs of the experiment (run 1:3). The chosen decoding parameters follow the recommendations of specialists in the field based on the previous literature on the use of the ML approach in analyzing ERPs, adapted to our data (Bae, 2021; Bae et al., 2020; Bae and Luck, 2018, 2019b,2019a; Baiano and Zeppieri, 2023; Carrasco et al., 2024; Grootswagers et al., 2017; Isik et al., 2014; Luck, 2023). Unlike the standard decoding of the continuous EEG signal, ERPs are specific due to the constraints of the paradigm (i.e., repetitions of the presented stimuli during EEG recording) and their nature as the brain’s responses to the upcoming stimuli. Therefore, since ERPs are the time series of electrical potential measured within a time window time-locked to the onset of the stimuli from the electrodes on the scalp, MVPA allows to decode of the pattern of voltages across the electrodes between the studied conditions. To this end, a separate support vector machine (SVM) classifier, a powerful and popular ML algorithm, was trained to discriminate between each class (here: run of the experimental session) and the other two runs based on the scalp distribution. Such an approach allowed the SVM to learn how the scalp distribution for the ERP from one run differed from the scalp distribution for ERP from the other runs at a particular time point (for each participant separately).

To take advantage of the excellent temporal resolution of the EEG signal, the classification was performed time point by time point for the whole ERP epoch and then investigated where, in time (relative to the stimulus onset), the decoding accuracy exceeded the chance level. Such an approach allowed us to study the dynamic brain activation pattern and compare the accuracies among the conditions.

Thus, the decoding for each participant was performed on the electric potential values measured at each variable: time (256 time points, from −200 to 800 ms time-locked to the stimulus onset), experimental run (run 1, run 2, run 3), trial (mean number of 48 ± 3 individual target trials, i.e., repetitions of the target stimuli, for each run), and electrode site (30 electrode channels). The number of time points resulted from the sampling frequency (256 Hz, i.e., 256 data points saved during EEG acquisition per second). Decoding was performed on the means calculated across the trials to improve the signal quality. A separate SVM classifier was then trained for each time point to discriminate between each run and the other two runs based on the scalp distribution. Therefore, a decision hyperplane (i.e., a hyperplane that separates two classes) through the 30-dimensional space was drawn (because there were 30 scalp channels) that separated the trials for each class. In other words, the classifier distinguished conditions based on voltage patterns across electrodes.

Therefore, each trial was a single point that represented the voltage value from a set of 30 channels. The SVM algorithm was used, as it tends to work well with a modest amount of data (Luck, 2023). The error-correcting output codes approach (ECOC) was used for decoding the ERPs among three classes, as recommended for multiclass decoding (Dietterich and Bakiri, 1995). One-versus-all decoding was used, in which each decoder learned to distinguish between one class and the other two classes. To test decoding accuracy, the data for a test case were fed into all the decoders, and the outputs of the decoders were combined into a single decision that provided the best guess for that case.

128 Hz effective sampling rate (i.e., every other time point) was used to increase signal quality (to minimize random fluctuations), which gave a temporal resolution of 8 ms. EEG (also ERPs) are time series, i.e., they are the values of voltage measured from the electrodes in time, using a pre-defined time step. This time step comes from the sampling frequency, which defines how many data points are being saved during data acquisition per time unit and determines the temporal resolution. E.g., the sampling rate of 256 Hz means there are 256 samples saved per second. This, in turn, means the time interval between the adjacent time points is 1/256 Hz ≈ 0.004 s = 4 ms. Therefore, offline subsampling to 128 Hz, by taking every other time point, results in having an 8 ms time interval between data points, i.e., the temporal resolution. Decoding was performed for the time range of the whole ERP epoch, which lasted 1 s, starting 200 ms before the onset of the stimulus, and ending 800 ms time-locked to the stimulus presentation. This length is appropriate to investigate late ERPs, such as P3. The details about the dataset’s structure are described in the Supplementary materials.

The important part of the classification is the cross-validation, i.e., training the algorithm on a subset of the data and then testing it on the data that was left out. To increase the precision, this procedure is usually repeated *k* times, each time leaving out a different subset for testing, therefore called a *k*-fold cross-validation. However, single-trial ERP data are noisy, which introduces a trial-to-trial variation. Thus, averaging multiple ERP trials is required. To fulfill these criteria, the data from each class were randomly divided into multiple subsets of trials, and each subset of trials was averaged separately. This led to having *k* folds x *n* trials per ERP for each class (e.g., 30 trials per condition gives 3 folds × 10 trials per ERP). In a standard ERP analysis on the subject level, all trials are averaged for each participant. However, since decoding needs validation, some portion of the trials needs to be left out. Therefore, all trials are divided into *k* folds (*k* averages), where each fold is an average of *n* trials. For instance, if there is a total number of 30 available trials (which is a typical number for most ERPs), they need to be equally divided. Since the minimal recommended number of trials per average is 10, this gives three-folds, where each fold is an average of 10 trials. Then, the classifier was trained on the *k*-1 averages (each having *n* trials) and tested on the remaining average. To improve the resolution of the decoding accuracy, this process was iterated, each time using a new random assignment of trials to averages (Luck, 2023). Here, the decoding was iterated 100 times, to balance between the calculation costs and obtaining sufficient decoding accuracy. The ERP pre-processing usually results in excluding a few trials that contain artifacts, so the final number of trials differed among the participants. Therefore, a common floor for the number of trials per ERP across all the subjects was used.

However, there are some technical aspects to consider in ERP research, e.g., the minimal number of trials (stimuli repetition) per condition, with 20–30 trials being acceptable for achieving satisfactory SNR in most cognitive ERPs (Luck, 2014). At the same time, the ML model requires sufficient input examples for proper cross-validation and generalization. Thus, the single-trial ERPs from each class were divided into subsets of trials. Previous studies have shown that the classifier performs well with 10 as the minimal number of trials per ERP and 3 as the minimal number of blocks for cross-validation (Bae, 2021; Grootswagers et al., 2017; Luck, 2023), which corresponds well with the typical 30 trials per condition in the ERP studies. However, there is still a decision to make when more trials are available, i.e., whether to increase the number of trials per ERP or the number of cross-validation blocks (where both increase the SNR at the single-subject level but exclude more subjects) or keep the minimal values of the decoding parameters but include more subjects, which results in increasing the statistical power at the group level.

Therefore, decoding was performed separately using three sets of decoding parameters to evaluate the effect of choice criteria to create the averaged ERPs (i.e., the number of trials averaged and the number of cross-validation blocks). First, 10 trials per ERP average and 3 cross-validation blocks were used, according to the recommendations on the minimal number of trials per ERP (set 1). Then, two more strict criteria at the subject level were used. One was focused on the number of trials per ERP average, i.e., 13 trials per average and 3 cross-validation blocks (set 2). The third set emphasized the number of folds, i.e., 10 trials per ERP average and 4 cross-validation blocks (set 3). These two criteria were chosen to use the available trials, considering the requirement of the common floor (i.e., the same number of trials) across all the participants. Therefore, from all 47 participants recruited, available were only those whose number of ERP trials after rejection was at least 30 (10 trials per ERP x 3 cross-validation blocks), 39 (13 trials per ERP x 3 cross-validation blocks), and 40 (10 trials per ERP x 4 cross-validation blocks) for sets: 1, 2, and 3, respectively. This resulted in including 31 participants for the MVPA using set 1, and 24 participants for the MVPA using sets 2 and 3.

### 2.6 Statistical analysis

#### 2.6.1 Decoding performance

A statistical analysis was performed on the decoding performance to evaluate whether the classifier successfully discriminated the ERP patterns among the experimental runs (classes) for each studied condition (placebo and study group). In decoding for interpretation, one does not need to obtain close to 1 decoding accuracy, as the goal is to increase the statistical power of the analysis and find differences between the analyzed conditions. Therefore, the classifier performs well if the decoding accuracy is significantly above the chance level. To this end, the decoding accuracies were compared against the chance level (0.33) for each condition separately (placebo and study groups) within the whole epoch (from −200 to 800 ms relative to the stimulus onset). In this study, the classes were the runs of the experiment: before the supplementation (run 1), 30 min after the supplementation (run 2), and 90 min after the supplementation (run 3). This gave a chance level of 0.33 because the classifier could classify the ERPs to one of the three classes. This classification was performed for each participant separately. Then, the decoding accuracies were averaged for the participants who were in the placebo group and for the participants who were in the study group, and the decoding averages were statistically compared against the chance level for each group separately using a one-sample permutation *t*-test with correction for multiple comparisons using the *mult_comp_perm_corr* function (Groppe, 2024), for each parameter set. This method adjusts *p*-values in a way that controls the family-wise error rate and is more powerful than the Bonferroni correction when different variables in the test are correlated. If the voltage pattern over the 30 electrodes contains information about the run, then the decoding accuracy should be greater than the chance level.

#### 2.6.2 Inter-group comparison

The decoding accuracies between the studied conditions (study and placebo groups) and parameter sets (set 1–3) were first averaged across 50-ms time bins along the ERP epoch to compare the decoding accuracies. Such a bin length allows to capture of possible effects in the time course of the studied ERPs and is often used in neurocognitive research to evaluate the studied effects along the ERP epoch. Then, a repeated measures ANOVA was used to compare these averaged accuracies (dependent variable) among the following categorical factors: condition (placebo vs. study group), parameter set (set 1, set 2, set 3), and time (200–250 ms, 250–300 ms, 300–350 ms, 350–400 ms, 400–450 ms, 450–500 ms, 500–550 ms, 550–600 ms, 600–650 ms, 650–700 ms). Shapiro-Wilk W test, Levene test, and Mauchley test were used to test the normality, homogeneity of variance, and sphericity, respectively. The data had a normal distribution and fulfilled the homogeneity of variance criterion. A Greenhouse-Geisser correction was used to correct violations of sphericity. *P* < 0.05 was regarded as statistically significant. Statistica v. 14.1 was used for this analysis.

#### 2.6.3 Comparison of the MVPA results with the previous standard and non-parametric cluster-based permutation analysis

A trend analysis was performed to compare the results obtained by the MVPA decoding with our results from the standard and non-parametric cluster-based analysis reported earlier.

Therefore, the hypothesis of this work is that the supplementation influences ERPs, which can be detected through decoding accuracy above the chance level. In addition, the effects that were not captured by the previously applied methods are expected to be observed using decoding.

## 3 Results

**FIGURE 2:**
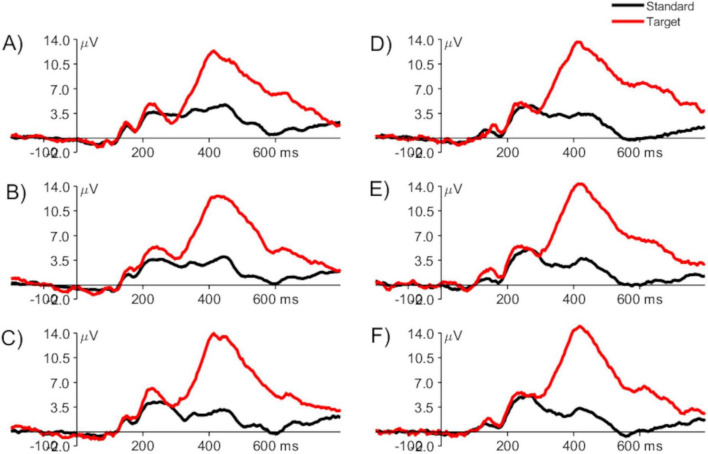
Grand averaged ERPs from the placebo group: run 1 **(A)**, run 2 **(B)**, run 3 **(C)**, and study group: run 1 **(D)**, run 2 **(E)**, and run 3 **(F)**, elicited by the standard (black) and target (red) stimuli, measured at channel Pz. The vertical line at time 0 ms represents the onset of the stimulus.

The dataset used for this study contained single-trial pre-processed ERPs elicited by targets from run 1, run 2, and run 3 of the visual P3 oddball task from the placebo and control groups. Figure 2 presents a representative grand-averaged ERP (i.e., averaged across all the participants) from runs 1 to 3 of the placebo and study groups, measured at channel Pz (parietal electrode at the midline).

The visual inspection of the ERPs shows a typical time course and characteristics of the waveforms, i.e., early sensory potentials, which seem not to change throughout the experiment in either condition, and much higher P3 for targets than for standards. This broad positive component is observed between around 300–700 ms after stimulus presentation, related to the attention process elicited as a result of the stimulus classification process (i.e., the participants had to evaluate whether each of the presented stimuli is a standard or a target, before deciding to push the button in reaction to the targets). P3 reaches a maximum around 350–550 ms after stimulus onset and is maximal over the central and parietal scalp areas. Its amplitude is related to attentional resource allocation and latency to the classification speed (Luck, 2014; Luck et al., 2000; Polich, 2007). More attentional resources directed to the target stimuli elicited a stronger P3 than the standard stimuli. In addition, smaller components (N1, P1, N2, and P2) are present and related to the sensory processing performed at the earlier stage.

**FIGURE 3:**
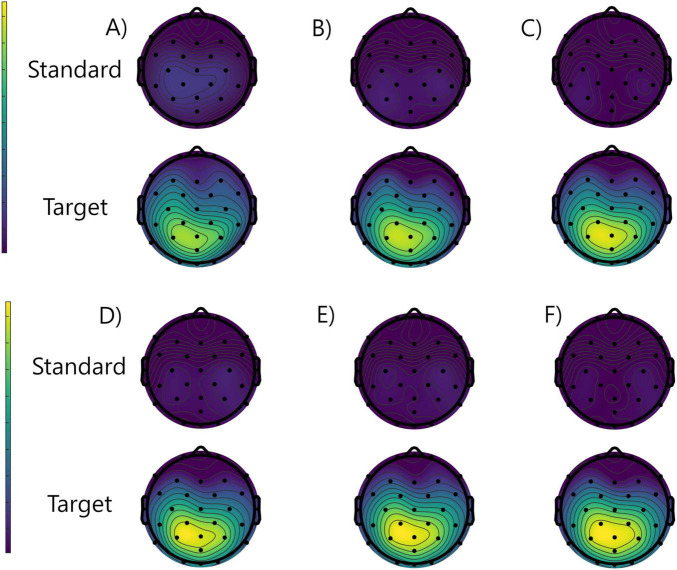
Grand averaged topographical distributions of the ERPs averaged between 350 and 550 ms from the placebo group: run 1 **(A)**, run 2 **(B)**, run 3 **(C)**, and study group: run 1 **(D)**, run 2 **(E)**, and run 3 **(F)**, for the standard (rows 1 and 3) and target (rows 2 and 4) stimuli.

To better visualize the scalp distribution of the broad P3 waveform in the studied conditions, the grand averaged topographical ERP distribution images that represent the ERP amplitudes averaged between 350 and 550 ms post-stimulus are presented in Figure 3. A posterior, midline distribution, typical for P3 component, is well seen (slightly higher in the left hemisphere) only in the trials time-locked to the targets, in both study and placebo conditions. Moreover, there is a slight tendency to increase its amplitude when going from run 1 to run 3 only in the placebo group.

### 3.1 Decoding performance

**FIGURE 4:**
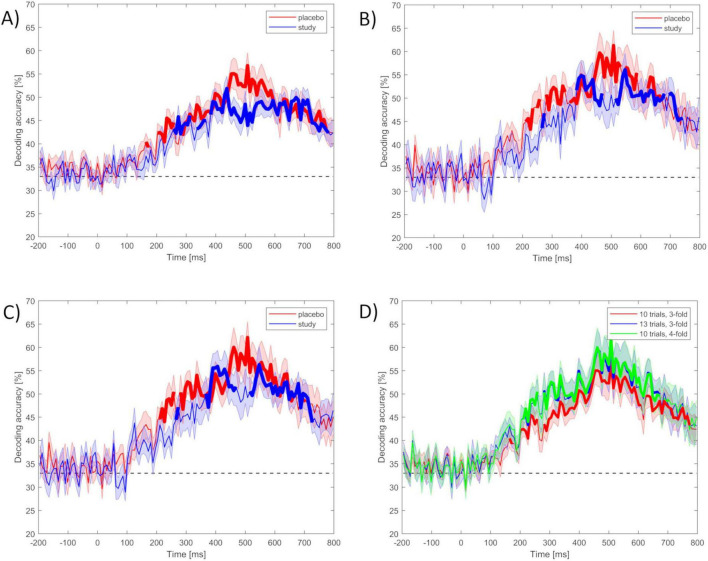
(**A–C)** Mean accuracy of ERP-based decoding performed using MVPA with the use of set 1–3 of decoding parameters among three classes (run 1, run 2, and run 3) for the placebo (red) and the study (blue) groups within the ERP epoch (- 200 to 800 ms): **(A)** set 1 (10 trials per ERP average and 3 cross-validation blocks), **(B)** set 2 (13 trials per ERP average and 3 cross-validation blocks), **(C)** set 3 (10 trials per ERP average and 4 cross-validation blocks). **(D)** Decoding accuracies from the placebo group among run 1, run 2, and run 3, using all three decoding parameter sets: set 1 (red), set 2 (blue), and set 3 (green). The dashed black line is the chance level (0.33). Stimulus onset is at 0 ms. Time intervals for which the accuracies were significantly above the chance level after correction for multiple comparisons are marked in bold lines.

The pre-processed ERP datasets from each participant were decoded at the subject level among the three runs (i.e., three classes) for the placebo and study groups separately. This gave the chance level = 0.33 (1 over 3 runs). First, the classification was performed using the minimal values of the decoding parameters (set 1), i.e., 10 trials per ERP average and 3 cross-validation blocks. The decoding accuracies calculated separately for each participant were then grand-averaged across all the participants from the placebo and the study group. Grand averaged decoding accuracies for the placebo and the study groups using set 1 are presented in Figure 4A.

Decoding accuracy for the ERP activity in the placebo group began to rise above chance around 200 ms after the stimulus onset, maintained at the highest level around 450–550 ms, with the maximal peak around 500 ms, and decreased as time after the stimulus increased. Time intervals for which the accuracies were significantly above chance level were: from around 200 to 260 ms and from 280 to 770 ms. These two intervals likely reflect the P2 (ERP component arising 200 ms after the stimulus onset, related to registration and early input classification) and P3 ERP components, respectively. Decoding accuracy for the ERP activity in the study group began to rise above chance later (around 260 ms). The maximal value was achieved around 400 ms, then dropped and remained at a similar level (lower than the placebo group) until 700 ms and then decreased again. Time intervals with decoding accuracy significantly above chance were from around 260 to 310 ms, from 335 to 370 ms, and from 380 to 780 ms.

**FIGURE 5:**
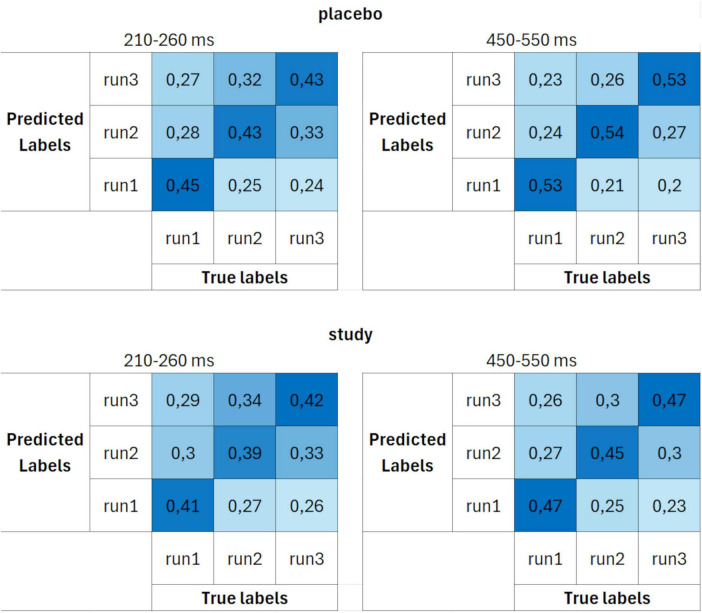
Confusion matrices calculated using parameter set 1 (10 trials per ERP average and 3 cross-validation blocks) for placebo (upper row) and study (lower row) groups, averaged over two time windows: 210–260 ms (first column) and 450–550 ms (second column). The units are proportion correct.

In addition to the time course of the ERP-based decoding accuracies, confusion matrices were calculated. Figure 5 presents confusion matrices for set 1 (10 trials per ERP average and 3 cross-validation blocks) for placebo and study groups, averaged over two time windows: 210–260 ms and 450–550 ms. Since decoding was performed for each time point within the ERP epoch, the confusion matrices have been constructed based on the average values within these two time windows, which represent the peaks of the two ERP components: P2 and P3. The confusion matrix is calculated at each time point, and it gives more information about the nature of the errors the decoder made. It shows the likelihood that a true class was classified as each of the alternative classes. For the purpose of this work, the confusion matrices were averaged over a time range that corresponded to the two ERPs of interest: P2 (210–260 ms) and P3 (450–550 ms). This was done to better visualize the decoding performance that yielded the best accuracy and was previously observed in Figure 4. Each cell shows the probability of a given classification outcome (y-axis, predicted labels) for a given class (x-axis, true labels), averaged over the two time intervals and across participants. The decoding showed a high probability of classification responses at the true values, as the classification responses were aligned around the central diagonal for both placebo and study groups. However, higher classification probabilities were achieved for the placebo group, especially for the 450–550 ms interval.

Figure 4B presents the time course of the decoding accuracies when the classification was performed using more strict criterion at the single subject level (set 2), i.e., the inclusion of 13 trials per ERP average, keeping the same number of cross-validation blocks (i.e., 3 blocks), which resulted in including 24 participants (12 in the placebo and 12 in the study group). Decoding accuracies had a similar time course as when set 1 was used. However, the difference between the placebo and study groups was much more pronounced here. The decoding accuracy in the placebo group rose above the chance level from around 210 ms after the stimulus onset, was maximal around 450–550 ms, and peaked around 500 ms, achieving almost 60%. Time intervals for which the accuracies were significantly above chance level were: from around 210 to 260 ms, from 280 to 340 ms, from 360 to 530 ms, from 550 to 600 ms, and from 630 to 670 ms. On the contrary, in the study group, it was significantly above chance only from around 380–470 ms and from 520 to 740 ms. It dropped considerably with the time interval of 450–500 ms (maximal value in the placebo group).

**FIGURE 6:**
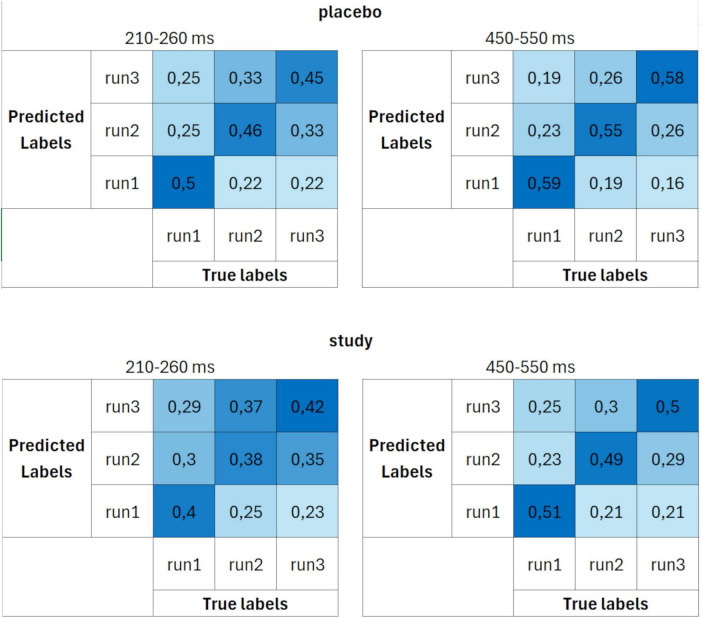
Confusion matrices calculated using parameter set 2 (13 trials per ERP average and 3 cross-validation blocks) for placebo (upper row) and study (lower row) groups, averaged over two time windows: 210–260 ms (first column) and 450–550 ms (second column). The units are proportion correct.

These differences between the placebo and study groups were further emphasized in the confusion matrices (Figure 6). The decoding performance was again better for the placebo than for the study group. However, the differences between the groups were higher than when set 1 was used. Moreover, whereas the decoding accuracies in the study group were similar for all three runs (around 40% for the 210–260 ms and around 50% for the 450–550 ms interval), in the placebo group, it was the highest for decoding run 1 (50% for the 210–260 ms and 59% for the 450–550 ms interval).

Figure 4C presents the decoding accuracies calculated using the last set of decoding parameters (set 3), i.e., 10 trials per ERP average and 4 cross-validation blocks. This resulted in 24 participants (12 in the placebo group and 12 in the study group). Here, the results were very similar to those obtained using set 2. Similarly, decoding accuracies above the chance level started around 210 ms after stimulus presentation for the placebo group and 270 ms after stimulus presentation for the study group. Time intervals for which the accuracies were significantly above the chance level in the placebo group were: from around 210 to 260 ms and from 280 to 670 ms. In the study group, it was mainly from around 370 to 470 ms and from 520 to 730 ms for the study group.

**FIGURE 7:**
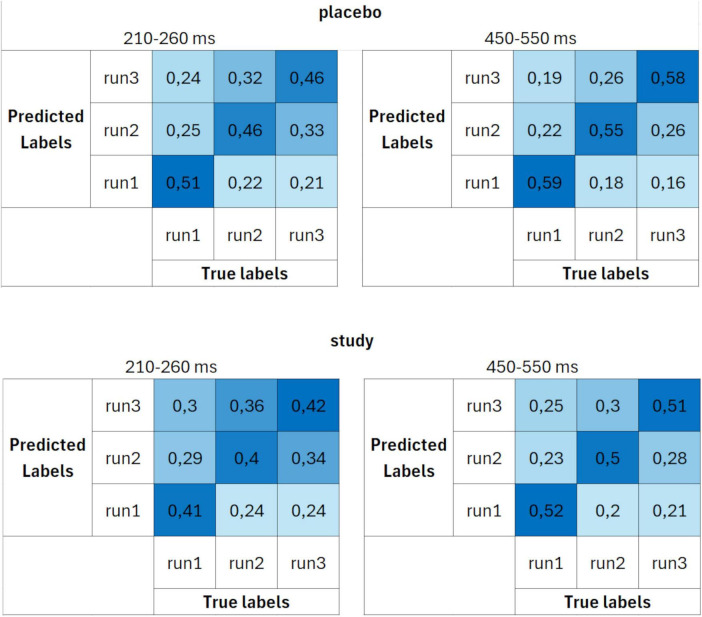
Confusion matrices calculated using parameter set 3 (10 trials per ERP average and 4 cross-validation blocks) for placebo (upper row) and study (lower row) groups, averaged over two time windows: 210–260 ms (first column) and 450–550 ms (second column). The units are proportion correct.

The confusion matrices calculated using parameter set 3 (Figure 7) had the same characteristics (and similar probabilities) as the ones for set 2. The decoding performance was again better for the placebo group than the study group, with larger differences than when set 1 was used. Again, the decoding accuracies in the study group were similar for all three runs, whereas they were the highest for decoding run 1 in the placebo group.

For all decoding parameters, decoding accuracies were at the chance level for the whole baseline time window, until around 200 ms after stimulus presentation (Figure 4). Time intervals for which the accuracies were significantly above the chance level for both the placebo and study groups are marked in Figure 4 in bold lines. Decoding accuracy was higher for the placebo than for the study group within around 450–590 ms post-stimulus, with maximum values of 0.57, 0.61, and 0.62 for parameters set 1, 2, and 3, respectively. This maximal decoding accuracy was achieved at 508 ms after stimulus onset for all parameter sets. In addition, for sets 2 and 3, decoding accuracy was higher in the placebo than in the study group, also within around 210 to 375 ms.

Figure 4D presents the decoding accuracies among all three parameter sets only for the placebo group. The above-chance decoding accuracy was achieved for all parameter sets within around 210–260 ms after stimulus onset, and then from around 280 to 770 ms for parameter set 1, and from around 280 to 670 ms for parameter sets 2 and 3. In addition, for both the placebo and the study groups, slightly higher decoding accuracies within these time ranges were obtained with parameter sets 2 and 3, compared to set 1.

### 3.2 Comparison of the decoding accuracies among the conditions and parameter sets

**FIGURE 8:**
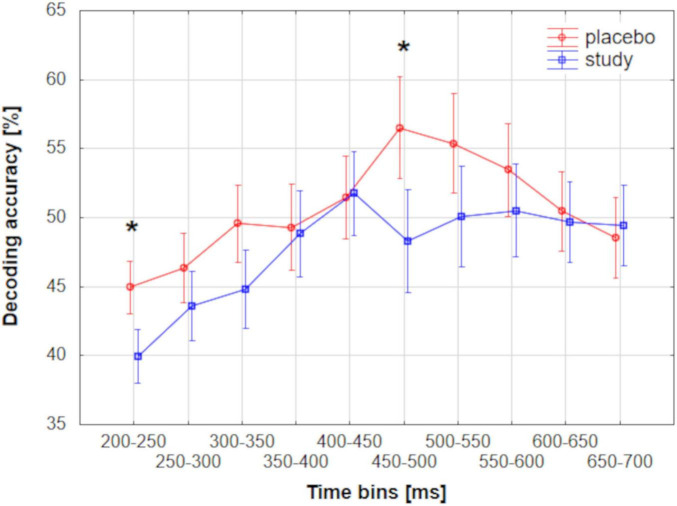
Mean group-level decoding accuracies performed using MVPA for placebo and study groups along the ERP epoch averaged across 50-ms time bins. Vertical bars represent 0.95 CI. Asterisks represent significant differences between the placebo and study groups.

To directly evaluate the differences in the decoding accuracies between the placebo and study groups and among the three parameter sets, the accuracies were averaged across 50-ms time bins between 200 and 700 ms and compared among the conditions. Such a time range was chosen because this analysis was targeted at the meaningful time range, so only the time bins that had a neurophysiological meaning were compared. Main effect TIME (F_9_, _657_ = 20.2, *P_*corr*_* < 0.001, partial eta-squared = 0.22) and interaction effect TIMExCONDITION (F_9_, _657_ = 3.7, *P_*corr*_* = 0.0037, partial eta-squared = 0.05) were significant. *Post hoc* tests revealed significant differences between the placebo and study groups for the following time bins: 200–250 ms (*P* = 0.035, Cohen’s d = 0.3) and 450–500 ms (*P* = 0.001, Cohen’s d = 0.7). Figure 8 presents mean decoding accuracies for placebo and study groups in the analyzed time bins.

#### 3.3 Trend analysis of the results obtained using standard methods, non-parametric cluster-based analysis, and MVPA decoding

A trend analysis has been performed to compare the results obtained in this work with our previously reported findings. Table 1 presents the effects studied using four methods: (1) evaluating the decoding performance against the chance level for the placebo and study groups, (2) comparison of the decoding accuracies between the studied conditions, (3) standard statistical testing, and (4) non-parametric cluster-based analysis. Methods 1 and 2 were performed in this work, whereas methods 3 and 4 have been described in Maciejewska and Grabowska (2020). Using the standard analysis, we found the main effect run, interaction effects RUNxGROUP (*post hoc* significant differences only in the placebo group), SESSIONxAREA (post-hoc significant differences over central and parietal areas) for the mean ERP amplitude averaged between 350 and 550 ms (i.e., related to the P3). The non-parametric cluster-based permutation analysis revealed one significant cluster (*P* = 0.003), which extended from approximately 400 to 520 ms over the centro-parietal area (Maciejewska and Grabowska, 2020). For clarity, among the ML results, only set 3 was included, as this set achieved the best performance. The comparison of the results using trend analysis was performed for three time intervals within the ERP epoch, which are neurophysiologically relevant: (1) early stimulus processing, related to the P2 component (∼200–260 ms), (3) post-processing related to the P3 peak (∼350–450 ms), and post-processing associated with the late part of the P3 component (450–550 ms).

**TABLE 1:** The results of trend analysis comparing the results between MVPA, standard analysis, and non-parametric cluster-based analysis.

ERP effect	MVPA			Standard analysis	Non-parametric cluster-based permutation analysis
	Placebo against chance	Study against chance	Significant difference in decoding accuracy between placebo and study group	main effect run, interaction effects RUNxGROUP	one significant cluster (*P* = 0.003)
Time window related to early processing: P2 component (∼200–260 ms)	↑↑	n.s.	↑↑	n.a.	n.s.
Time window related to post-perceptual processing: maximum of P3 component (∼350–450 ms)	↑↑	↑↑	n.s.	↑↑	↑↑
Time window related to post-perceptual processing: late part of the P3 component (∼450–550 ms)	↑↑	n.s.	↑↑		

↑↑ means statistically significant (*P* < 0.05), n.s. means non-significant, n.a. means not applicable.

Analyzing the data in Table 1, we can see that the experimental effect in the early time window (∼210–260 ms) was obtained only when ML was used. First, the above-chance decoding in this time window was observed only in the placebo group. Further ANOVA analysis confirmed a significant difference in the decoding accuracy between the placebo and study groups in this time bin. The standard univariate analysis could not test the studied effect in this time window because it used a different time window chosen *a priori*. Non-parametric cluster-based analysis did not reveal a cluster corresponding to this time window.

Within the time window related to the P3 peak (∼350–450 ms), the decoding was above chance for both the placebo and study groups, and there was no significant difference in the decoding accuracies in this time window. The standard analysis revealed a significant difference in the P3 amplitude between the placebo and study groups. However, this difference was calculated for the *a priori* time window of 350–550 ms, so it is impossible to conclude about only a part of such a window.

All methods revealed a significant effect within the time window related to the later part of the P3 component (∼450–550 ms). The decoding accuracy was above chance only in the placebo group, and the ANOVA analysis confirmed this difference. The standard analysis revealed a significant difference in the decoding accuracies between the placebo and study groups within the 350–550 ms. Finally, non-parametric cluster-based analysis showed a significant cluster corresponding to the 400–520 ms.

## 4 Discussion

The goal of this study was to decode the acute effect of energy supplementation on the ERPs elicited in a visual oddball paradigm, i.e., a cognitive task engaging attentional resources. MVPA was used to dissociate the ERPs among three runs: (1) before, (2) 30 min after, and (3) 90 min after digestion of a single dose of an energy dietary supplement with a small amount of caffeine (study group) or a placebo (placebo group) among young, healthy volunteers.

### 4.1 Decoding the impact of energy boost supplementation

In our previous work (Maciejewska and Grabowska, 2020), we found a significant increase in P3 amplitude, defined as a mean amplitude between 350 and 550 ms, throughout the experiment (run 1 - > run 2 - > run 3) only in the placebo group, over central and parietal areas. However, the standard univariate testing has several limitations, such as high data dimensionality, selection of the range of time and channels included in the analysis, and MCP. Such an approach may lead to the loss of effects that have not been found yet or that are too small (Cohen, 2014). Therefore, to overcome this problem, in the next step of the same work, we used non-parametric cluster-based permutation analysis, where the univariate tests are performed point-by-point, corrected for MCP, and spatiotemporal clusters are created (Maris and Oostenveld, 2007). In the analysis, all time points (i.e., the whole epoch) and all 30 electrodes were included in the permutation test. The analysis revealed one significant cluster (*P* = 0.003, cluster corrected), which corresponded to a broad positive spatiotemporal cluster from around 400 to 520 ms over the centro-parietal area (Maciejewska and Grabowska, 2020). However, this approach is based on each subject’s averaged ERPs, which don’t consider the intra-trial variability across the single recording.

Therefore, in the current work, an alternative method was used, MVPA, to better discriminate the voltage pattern across electrode sites. First of all, the ERPs were successfully decoded among three runs of the experiment for both the placebo and the study group (observed as the above-chance decoding accuracies marked in bold in Figures 4A–D). To interpret the neurophysiological underpinnings of these results, the time course of the decoding performance within the ERP epoch will be discussed within the three time intervals, each related to different brain processing stages.

First, the decoding accuracies were at the chance level (i.e., 0.33) for the whole baseline time window, i.e., between −200 to 0 ms, for both the placebo and the study group, regardless of the parameter set used (*P* > 0.18 for both groups and all sets). This is physiologically valid since the baseline time window represents the time before the stimulus presentation, i.e., without task-related brain activity. This information is crucial because it serves as an additional validation of the decoding performance in terms of the physiological signal. Since the pre-stimulus period is related to the ongoing brain activity unrelated to the task, we should not expect decoding accuracy above the chance level in the baseline time window. This confirms that there are no differences in the analyzed ERPs among the classes that are not physiological.

The second time interval is between the onset of the stimulus (i.e., 0 ms) and around 200 ms. This interval represents the time when the visual stimulus is processed by the visual pathway from the retina (which is not captured by EEG) to the primary visual cortex (calcarine cortex) (Luck and Kappenman, 2012). Visual evoked potentials (VEPs) are the first early sensory potentials elicited in the visual modality. They are related to the physical parameters of the stimulus but are independent of the consciousness and attention state of the participant. The most stable VEP is P1, a positive component with a maximal amplitude of around 100 ms, preceded by N1 at around 75 ms and followed by N2 at around 140 ms (Baiano and Zeppieri, 2023). The decoding accuracies within this time window were higher than from the baseline (especially when sets 2 and 3 were used). However, they did not reach a significance level above chance for either the placebo or the study group (with the lowest *P* within the 0-200 ms of: 0.32, 0.49, 0.27, 0.45, 0.36, and 0.47 for: placebo group from set 1, study group from set 1, placebo group from set 1, study group from set 1, placebo group from set 1, study group from set 1, respectively). This means that the classifier failed to distinguish the ERPs in this time interval among the three classes (runs). These components are related to the physical characteristics of the stimuli. In this study, the physical parameters of the stimuli were kept the same across the compared runs and conditions. Thus, these results confirm that the early neural processing of the visual stimuli (from around 100 to 200 ms) is not influenced by the increasing mental fatigue elicited in this paradigm, nor affected by the single dose of caffeinated energy boost dietary supplement. In some paradigms that focus on the early attentional processes, there are early attentional processes that are manifested in the ERPs as the P1 suppression for the stimuli at unattended locations (i.e., a location on a screen where the participant is not paying attention to) and N1 enhancement at attended locations (i.e., a location on a screen where the participant is focusing, e.g., the location where the target stimuli are presented). These effects are related to visuospatial attention, i.e., when the stimuli that the participants observe are presented in different locations of the visual field. Here, however, all the stimuli were presented in the center of the screen, so there was no competition between spatially distinct information streams.

The decoding accuracy started to rise above the chance level around 200 ms post-stimulus. However, at this stage, differences between the placebo and study groups became apparent. In the placebo group, the accuracy was significantly above the chance level as early as around 200–260 ms after stimulus presentation (with the lowest *P* < 0.001 for all sets), whereas only after 260 ms in the study group (with the lowest *P* of: < 0.001, 0.014, and 0.014, for sets 1–3, respectively). This discrimination between the conditions is especially well observed where sets 2 and 3 of the decoding parameters were used (Figures 4B,C). In those cases, the above-chance decoding accuracies in the study group were achieved only after around 370 ms (with the lowest *P* of: < 0.001 for both sets), except for a short time interval of less than 10 ms, which, considering the decoding accuracy of 8 ms, is not physiologically relevant. The statistical comparison of the decoding accuracies for 50-ms time bins between the conditions confirmed that it is significantly higher in the placebo than the study group in this time window with *P* = 0.035 and Cohen’s d = 0.3 (200–250 ms time bin, Figure 8). This time window is related to the P2 component which is present later than the fast, purely sensory components, but earlier than higher cognitive waveforms, such as P3 (Figure 2). It is generated around 200–260 ms and represents, among others, the registration and early input classification, early selective attention, and detection of stimulus features (Dunn et al., 1998; Michalkova et al., 2022; Xia et al., 2018). The fact that the classifier successfully distinguished the ERPs in this time interval among the runs only in the placebo group means that the P2 amplitude significantly changed from run 1 to run 3 in this condition. On the other hand, it remained at a similar level in the study group. Indeed, in Figure 2, one may observe an increasing P2 amplitude time-locked to the target when going from run 1 to run 3 in the placebo group. Whereas in the study group, it remains at a similar level and is not distinguishable from the standard-related trials. This pattern of results is also visible when looking at the confusion matrices (Figures 5–7). Mean decoding accuracies averaged across 210–260 ms (corresponding to the P2 component) tend to be higher in the placebo than in the study group for all parameter sets. Moreover, the highest values were obtained for run 1, which can be interpreted as the easiest to decode, i.e., differing more from the other two runs. This is in line with the ERP traces, which show a higher increase between run 1 and run 2 than between run 2 and run 3 (Figures 1, 2 from Maciejewska and Grabowska, 2020). Indeed, ERP literature has shown that featural attention may be manifested as an anterior P2 attention effect (anterior selection positivity) (Luck, 2014; Luck and Kappenman, 2012). This type of attention effect is related to the processing of non-spatial features. The fact that the decoding accuracies were higher for the placebo than for the study group in this time interval suggests that the dietary supplement affected the early classification, attention, and detection of stimulus features processes.

Finally, the decoding performance was the highest within the last, broad, and late time window. It was higher for the placebo than for the study group within around 450–590 ms post-stimulus (regardless of the decoding parameters). It was maximal (62%) when parameter set 3 was used. In this time window, a P3 is observed, originating from temporal-parietal activity associated with attention (Luck, 2014; Polich, 2007). In an oddball paradigm, P2 and P3 are larger for targets than standards, but the P2 effect occurs only when simple stimulus features define the target. At the same time, the P3 effect can also be observed for complex target categories (Luck, 2014). Interestingly, the decoding accuracy in the study group did not achieve above-chance performance within the P3 time window, i.e., from around 470 to around 520 ms, when sets 2 and 3 were used (*P* > 0.069 and *P* > 0.057 for set 2 and 3, respectively, Figure 4). This discrimination between the conditions was confirmed by the statistical comparison of the decoding accuracies for 50-ms time bins between the conditions. The decoding accuracy was significantly higher in the placebo than in the study group in the 450–500 ms time bin (*P* = 0.001, Cohen’s *d* = 0.7). The interpretation is the same as in the P2 component, i.e., the classifier successfully distinguished the ERPs in this time interval among the runs only in the placebo group because the P3 amplitude significantly changed from run 1 to run 3 in this condition, whereas it stayed at a similar level in the study group. This pattern of results is also visible when looking at the confusion matrices (Figures 5–7). Mean decoding accuracies, averaged across 450–550 ms, i.e., the time corresponding to P3 with the highest accuracy, tend to be higher in the placebo than the study group for all parameter sets. The highest values were obtained for run 1 for sets 2 and 3 (59%), like the results in the P2 time interval. These results support the ones reported by us earlier (Maciejewska and Grabowska, 2020). The differences in the time intervals for which the significant results were obtained using the three analysis methods result from the methodological differences of these analyses. The standard analysis uses an *a priori* time window, so if the time window was specified at 350–550 ms, one cannot infer when exactly the difference occurred within this time interval. On the other hand, non-parametric cluster-based analysis allows only for testing whether or not there was a significant difference within the analyzed epoch and to relate the effect to a spatiotemporal cluster (Maris and Oostenveld, 2007). Therefore, the time interval that corresponds to the effect is approximate.

Importantly, the output from all the analyzed methods shows a significant difference in how the P3 changed throughout the runs between the placebo and study groups, which started probably after 400 ms and lasted around 100 ms. These results indicate that a single dose of energy supplementation with a small amount of caffeine inhibited an increase in the P3 amplitude throughout the experimental session. P3 amplitude depends on uncertainty, probability, and resource allocation (Luck, 2014). However, the uncertainty and probability did not change between the runs and conditions in our experiment. Therefore, the increase in P3 amplitude among the runs in the placebo group seems to be an effect of increased resources allocated to perform the task. This increase in P3 probably results from increasing mental fatigue caused by having to complete many tasks during a long experimental time. This interpretation is consistent with our results on resting-state EEG from the same experiment, where we observed an inhibition of an increase in low-frequency brain oscillations caused by the supplementation (Maciejewska and Moczarska, 2023). Clinical data suggest that attention-related ERPs are linked to dopaminergic, norepinephrine, and catecholaminergic activity, with the involvement of the locus coeruleus–norepinephrine (LC-NE) system and acetylcholine (Ach) receptors (Burk et al., 2018). The energy-boost supplements impact the CNS by blocking the adenosine receptors A1 and A2, which prevents adenosine metabolism and results in increased dopamine and norepinephrine activity through releasing catecholamines. This mechanism explains how such supplementation may influence ERPs as the neurophysiological representation of attention processes. These results point out the necessity of controlling the uptake of dietary supplements before the neurophysiological examinations.

Overall, this part of our data showed higher decoding accuracies in the placebo than in the study group, in time intervals related to P2 and P3 ERPs. Our results indicate that the ERPs in participants who drank the supplementation changed throughout the experimental session considerably less than those from the placebo group. This pattern of results suggests that the dietary supplement inhibited the increasing changes in the ERPs, related probably to the progressive mental fatigue in the participants, as described by us earlier (Maciejewska and Grabowska, 2020; Maciejewska and Moczarska, 2023), and as suggested by previous findings (White et al., 2017).

Such a neurophysiological state of mental fatigue was purposely induced by us to simulate a real-world situation in which someone needs an energy boost when feeling tired and mentally exhausted. Noteworthy, the current work shows that such a supplementation-elicited inhibition affected not only the post-perceptual attention process, but also the early classification, attention, and detection of stimulus features processes already at the early processing stage. It is worth noting that, while using the traditional ERP analysis, the individual differences considerably impact the ERPs, decoding is performed on a single-subject level. This makes decoding more sensitive and allows detection of effects that may not be visible in the standard analysis.

### 4.2 Comparison of the decoding criteria

In the next part of the analysis, the decoding accuracies were compared among the three choices of decoding criteria. Previous research on ERP decoding suggests that the minimal requirements for a good classification performance of ERPs are 10–20 trials per ERP average and three-fold cross-validation. This considers the limitation of the total number of trials discussed in the Materials section. Averaging single-epoch ERPs is necessary for analyzing ERPs because it enhances brain responses to the stimulus while reducing random fluctuations of the ongoing activity not related to the stimulus processing. It reduces random variability and the overlap between the set of points for each class. The benefit of decoding is that it is done at the single-subject level. Thus, it masks the potential individual differences to a lesser extent. That is why this method is more sensitive. However, the important step is to choose the optimal number of folds and trials per fold. A higher number of folds increases the number of training cases but also reduces the SNR of the ERPs, as fewer trials are being averaged. Another thing is that including more trials results in excluding more participants at the group level of the analysis, since a different number of trials per subject survives the pre-processing of the EEG signal. In addition, when the decoding accuracy is compared between different groups, the number of trials needs to be floored (i.e., equate to the dataset with the smaller number of trials per ERP), as its differences may introduce differences in decoding accuracy. This issue was the goal of the second part of this work.

The comparison of the decoding accuracies among the three sets of decoding parameters did not reveal significant differences (*F*_2_, _73_ = 2.0, *P* = 0.15, partial eta-squared = 0.051). However, even though there was no SET effect when comparing the three parameter sets, sets 2 and 3 tend to perform better within 210–400 ms and 450–650 ms time intervals (Figure 4D). The confusion matrices also suggest higher performance when sets 2 and 3 were used compared to set 1 (Figures 5–7). This trend suggests the greater importance of having a better SNR at the subject level (i.e., more total number of trials per subject) than having more participants included at the group level. In addition, since the decoding accuracies were comparable between sets 2 and 3, it doesn’t matter whether there are more trials per condition or more folds. Such a pattern of results agrees with the fact that decoding is more sensitive than the traditional approach because it operates at the single-subject level.

However, since these results show only trends with no significant differences, the impact of the number of trials per ERP vs. the number of cross-validation blocks vs. the number of participants needs further investigation.

To sum up the overall discussion, this work shows that even a single dose of a dietary supplement that has much less caffeine than a cup of coffee changes brain physiology in mental fatigue. In cognitive neuroscience, when the studies are performed with human participants or patients, there are usually several instructions provided to the participants regarding what should or should not be done before the examination, such as not consuming alcohol or other substances that might influence the activity of the brain. However, these procedures are not uniform and depend on the research laboratory. The current study shows that even products that contain only a small amount of caffeine may change the brain’s activity, manifested in the early and later stages of processing visual stimuli. This points out the necessity of controlling the uptake of such supplements before the neurophysiological examinations.

Moreover, the significance of this study for a general audience lies in its applications to cognitive enhancement and public health. First, it shows the potential benefit of such multi-ingredient supplements for healthy users in increasing alertness and energetic arousal, improving psychomotor and mental performance, including memory and attention, and decreasing mental fatigue and stress. However, it might also be a valuable tool for patients suffering from cognitive impairment and mental disorders, such as Alzheimer’s disease, Parkinson’s disease, depression, and anxiety. In such cases, multi-ingredient supplementation might aid traditional treatment protocols related to attention, anxiety, mood, and memory disorders.

## 5 Conclusion

Our results show several key points. First, the ERPs were successfully decoded among three classes, i.e., three runs of EEG recorded before (run 1), 30 min after (run 2), and 90 min after (run 3) acute energy boost dietary supplementation with a small amount of caffeine. The above-chance decoding accuracy was significantly higher for the placebo than the study group within the time window of P2 and P3 components (200–250 ms and 450–500 ms time bins), showing that ERP amplitudes increased more in the placebo than in the study group. This indicates the change of the attentional resources toward the target stimuli throughout the experiment and that this change was higher for the placebo than for the study group, which reveals the effect of single-dose supplementation on ERPs elicited in a cognitive task engaging attentional resources. Although the supplement had a much smaller amount of caffeine than in a cup of coffee, it inhibited the increase of mental fatigue throughout the experimental session. Observing the effects in P2 and P3 time windows indicates that the studied effect impacts both the early and late stages of attention. These results extend our previously reported findings, which showed this effect only in the P3 time window. This emphasizes the value of decoding for interpretation in ERP research. A comparison among three sets of decoding parameters, though it did not reveal significant results, suggests the greater importance of having a better signal-to-noise ratio at the subject level (i.e., more trials per ERP average or more folds) than having more participants included at the group level. The issue of the optimal number of trials per ERP/folds/participants, however, needs further investigation. The significance of this work lies in showing that only a single dose of a dietary supplement that has much less caffeine than a cup of coffee changes brain physiology in mental fatigue. This points out the necessity of controlling the uptake of such supplements before the neurophysiological examinations. In addition, the results allow translation into clinical applications focused on pathophysiology related to attention, anxiety, mood, and memory disorders.

## Data Availability

The datasets presented in this study can be found in online repositories. The names of the repository/repositories and accession number(s) can be found at: https://osf.io/9tn5q/, OSF, DOI: 10.17605/OSF.IO/9TN5Q, https://github.com/kariniak/Decoding-the-effect-of-an-energy-dietary-supplement-on-visual-P3_public.
