# Supplementary material for: Decoding event-related potentials: single-dose energy dietary supplement acts on earlier brain processes than we thought
Source: Front Neuroinform. 2025 Jul 8;19:1563893. doi: 10.3389/fninf.2025.1563893 (PMC12279804; doi:10.3389/fninf.2025.1563893)
Supplement: Supplementary file 1 [file Table_1.docx]

Supplementary materials

An important feature of the EEG data is its high dimensionality, related to the number of recording channels, sampling rate, repetitions of the stimuli presented to the participants, number of conditions or groups, and sample size of the participant's groups. In this study, the single dataset from one participant and one run contained the values of the voltage recorded from 30 scalp channels, which is typical in cognitive neuroscience. However, 64 or 128 channels are also commonly used (Luck, 2014). The sampling rate was 256 Hz (which means data points being saved every 3.91 ms, i.e. 256 data points per second), which is also a standard value due to the Nyquist criterion, according to which the sampling frequency should be more than twice the maximal oscillation frequency of the measured signal. Higher cognitive processes (such as attention) are related to the neural oscillations of up to around 100 Hz (typically less), however much higher sampling frequencies are also used in some applications, such as somatosensory potentials or high-frequency oscillations (HFO), where fast, sensory processing may be up to 500 Hz (Park & Hong, 2019). Another important factor is the epoch length, i.e. the time window of the ongoing EEG extracted from the signal, time-locked to the stimulus onset. This depends on the latency of the studied ERPs. Each epoch contains a baseline, which is a piece of the signal recorded a few hundred milliseconds before the onset of the stimulus, and then its mean value (typically around 100-300 ms) is subtracted from each data point post-stimulus in the process of baseline correction (Luck, 2014; Polich, 2007). Here, the epoch was from -200 to 800 ms, which, having a 256 Hz sampling rate, gives 256 data points per epoch. Attention-related ERPs are typically elicited in an oddball paradigm, where the participant is presented with a series of stimuli from two categories: standard (around 80% of all the trials) and targets (20% of all trials), and is instructed to focus their attention on the targets (Polich, 2007). In this study, only targets were analyzed and their number before the data pre-processing was 60. Taking all these factors into account, the dataset size in this study is 30 channels x 256 data points x 60 trials x 3 runs x 47 participants.
